# A MoS_2_ Nanosheet-Based Fluorescence Biosensor for Simple and Quantitative Analysis of DNA Methylation

**DOI:** 10.3390/s16101561

**Published:** 2016-09-22

**Authors:** Le Xiao, Li Xu, Chuan Gao, Yulin Zhang, Qunfeng Yao, Guo-Jun Zhang

**Affiliations:** 1 School of Laboratory Medicine, Hubei University of Chinese Medicine, 1 Huangjia Lake West Road, Wuhan 430065, China; lexiao1993@163.com (L.X.); cgao@hbtcm.edu.cn (C.G.); Zhangyulin@whu.edu.cn (Y.Z.); 2 School of Pharmacy, Hubei University of Chinese Medicine, 1 Huangjia Lake West Road, Wuhan 430065, China; xuli81819009@126.com

**Keywords:** MoS_2_, fluorescence biosensor, homogeneous analysis, DNA methylation

## Abstract

MoS_2_ nanomaterial has unique properties, including innate affinity with ss-DNA and quenching ability for fluorescence dyes. Here, we present the development of a simple fluorescence biosensor based on water-soluble MoS_2_ nanosheets and restriction endonuclease BstUI for methylation analysis of p16 promoter. The biosensing platform exhibited excellent sensitivity in detecting DNA with a linear range of 100 pM~20 nM and a detection limit of 140 pM. More importantly, our method could distinguish as low as 1% difference in methylation level. Compared with previous methylation analysis, our design is both time saving and simple to operate, avoiding the limitations of PCR-based assays without compromising performance.

## 1. Introduction

The process of DNA methylation is an essential part of epigenetics that plays critical roles in many biological events, such as gene transcription, X-chromosome inactivation, and genomic imprinting [1,2,3]. In mammals, DNA methylation occurs mainly in CpG-rich regions, known as CpG islands, which is usually located in the promoter region or near the first exon of transcriptional regulatory genes [4,5,6]. Aberrant methylation of CpG islands was proven to be closely associated with the occurrence of human diseases, particularly cancers [7,8]. The initiation of a tumor is often accompanied by abnormal rise of methylation levels of CpG islands near tumor suppressor genes, which leads to their inactivation [6,7,8]. Thus, the change of methylation status in CpG islands is considered to be a promising biomarker for cancer prognosis and diagnosis [9,10,11].

Due to the great significance of research on DNA methylation status, a variety of biosensors and bioassays have been established for the quantification of gene-specific CpG methylation. Techniques based on bisulfite treatment and polymerase chain reaction (PCR) are the most extensively applied for the detection of methylation status [12,13,14]. However, these methods involve complicated procedures and require precision instruments. In addition, frequent false positive detection has become their common bottlenecks [15,16]. Lately, some new biosensing technologies without bisulfite or PCR for gene-specific methylation assays have also been developed, such as nanowire field effect transistor (FET) [16], electrochemistry [17], colorimetry [18] and so on. For example, Maki et al. [16] immobilized monoclonal anti-5-methylcytosine antibodies on the nano-FET, which could recognize and bind to methylated target DNA. Dai et al. [17] designed a label-free electrochemical DNA biosensor for quantification of gene-specific methylation, in which the probe was modified on gold electrode and methylene blue (MB) was used as the electrochemical indicator. Although these methods each have its advantages, they generally require tedious preparation work, such as sensor surface modification or extra amplification technology. Therefore, it is still necessary to explore convenient methods for the detection of gene-specific methylation.

Currently, nanomaterials are particularly useful in the field of biosensors due to their unique optical properties. For example, MoS_2_ nanosheets exhibit high quenching efficiency to fluorescence probes [19,20,21]. What’s more, as an emerging class of alternative graphene-like 2D nanomaterial [22], MoS_2_ nanosheets have demonstrated their intrinsic discrimination abilities to ss-DNA and ds-DNA, with even better water solubility [19,20,21,22,23]. Thus, combination with fluorescent DNA probes, a few MoS_2_-based biosensors has been developed for the detection of biomolecules, such as nucleic acids [19,20,21], proteins [24], and small molecules [25]. However, such a great biosensing platform has yet been employed for gene-specific CpG methylation analysis.

**Scheme 1:**
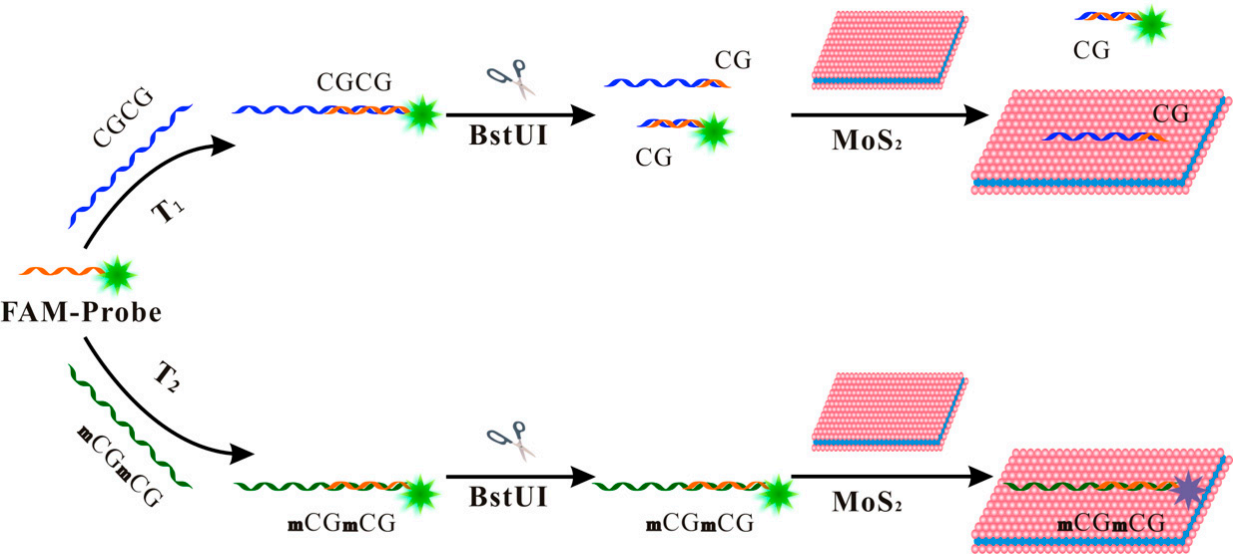
Schematic illustration of homogeneous methylation analysis of p16 promoter by means of MoS_2_ nanosheets-based biosensor.

We herein report a MoS_2_-based fluorescence biosensor for methylation analysis of p16 promoter with easy and quick operation. The mechanism of the sensing system is depicted in Scheme 1. A segment from the promoter of the p16 gene is selected as the investigated target, which includes the recognition site of BstUI restriction endonuclease. The FAM-probe (P) is firstly hybridized with unmethylated and methylated target DNA (T_1_ and T_2_) to form partial duplex (pds-DNA), respectively, and then mixed with BstUI. At last, MoS_2_ nanosheets are added into the hybridization solutions. As a result, the unmethylated pds-DNA (P/T_1_) is cleaved at specific site 5’-CGCG-3’ and FAM-labeled ds-DNA is released to the solution. Meanwhile, the methylated pds-DNA (P/T_2_) is adsorbed by MoS_2_, which lead to quenching of fluorescence. Thus, we provide a straight-forward approach to quantifying methylated DNA through fluorescence detection in homogeneous solution.

## 2. Materials and Methods 

### 2.1. Materials and Reagents

The restriction endonuclease BstUI was obtained from New England Biolabs (Beverly, MA, USA). The layer molybdenum disulfide (MoS_2_) nanosheets solution (1–8 monolayers, 100–400 nm, 18 mg/L) was purchased from Nanjing XFNANO Materials Tech Co. Ltd. (Nanjing, China) and treated by ultrasonic agitation for 3 h before use. Tris(hydroxylmethyl)aminomethane (Tris), sodium chloride (NaCl) and magnesium chloride (MgCl_2_) were purchased from Sinopharm Chemical Reagent Co. Ltd. (Shanghai, China). All the chemical reagents were of analytical reagent grade and used without further purification. Ultrapure water used in all experiments was generated from a Milli-Q Direct 8 water purification system (Millipore, Billerica, MA, USA).

The buffer solutions employed in this work were as follows: DNA hybridization buffer and BstUI reaction buffer were 1 × CutSmart buffer (50 mM potassium acetate, 20 mM Tris-acetate, 10 mM magnesium acetate, 100 µg/mL BSA, pH 7.9, 25 °C). MoS_2_ quenching and fluorescence detection were performed in buffer containing 20 mM Tris-HCl, 100 mM NaCl and 10 mM MgCl_2_ (pH 7.4).

All DNA sequences used in this work were synthesized by Sangon Biotechnology Co. Ltd. (Shanghai, China) and dissolved in ultrapure water to 100 µM stock solution and stored at −20 °C. Gene sequences of probes and targets include methylated and unmethylated were designed according to the promoter region human p16 gene. Non-complementary DNA (N) was from the promoter region of human p53 gene [26]. Detailed sequences are shown in Table 1.

### 2.2. Apparatus

All fluorescence measurements were carried out on a F-4600 spectrophotometer (Hitachi Co. Ltd., Tokyo, Japan) equipped with a xenon lamp as excitation source. The apparatus for transmission electron microscopy (TEM) was a JEM-2100 (Jeol Ltd., Tokyo, Japan). The UV-vis spectra were obtained using a UV-2550 spectrometer (Shimadzu, Tokyo, Japan). Atomic force microscopy (AFM) image was obtained from a multimode VIII (Veeco, New York, NY, USA). The dynamic light scattering (DLS) was conducted by a Zetasizer Nano ZS (Malvern Instruments Ltd., Malvern, UK)

### 2.3. Endonuclease Digestion of Probe/Target DNA

20 nM FAM—labeled probe DNA(P) were hybridized with unmethylated target DNA (T_1_, 20 nM) and methylated target DNA (T_2_, 20 nM), respectively. The mixture were heated to 95 °C for 10 min, and then slowly cooled down to room temperature to ensure the formation of partial duplex DNAs (pds-DNAs), including the unmethylated pds-DNA (P/T_1_) and the methylated pds-DNA (P/T_2_). Then the pds-DNAs were cleaved by 20 U/mL BstUI endonuclease at 60 °C for 2 h in 50 µL 1 × CutSmart buffer. Finally, the mixture pds-DNAs were mixed with 450 µL solution of 4 μg/mL MoS_2_ for 10 min, respectively.

### 2.4. Fluorescence Assays

Fluorescence measurements were carried out at room temperature. The emission spectra are measured in the range between 510 and 650 nm for carboxyfluorescein (FAM) with the excitation wavelength set at 495 nm.

### 2.5. Methylation Assay by Gel Electrophoresis

Non-denaturing polyacrylamide gel electrophoresis (PAGE) was used to verify the feasibility of the sensing system. In the gel electrophoresis experiment, 1 µM pds-DNAs (P/T_1_ or P/T_2_) were prepared and the specimens were treated as follows: (1) unmethylated pds-DNA (P/T_1_); (2) methylated pds-DNA (P/T_2_); (3) unmethylated pds-DNA (P/T_2_) and BstUI; (4) methylated pds-DNA (P/T_2_) and BstUI. The DNA solutions mixed with 1 × loading buffer were loaded on a 15% non-denaturing polyacrylamide gel electrophoresis. The electrophoresis was run at 100 V constant for 105 min in 1 × TBE running buffer (89 mM Tris-Boric Acid, 2.0 mM EDTA, pH 8.3). Subsequently, the gel was stained by ethidium bromide for 15 min, and then de-stained in ultrapure water for 15 min. Finally, electrophoresis images were captured using a Gel Doc XR+ imaging system (Bio-Rad, Hercules, CA, USA).

## 3. Results

### 3.1. Characterization of MoS_2_

The MoS_2_ was characterized and the results are shown in Figure S1. The transmission electron microscopy (TEM) image showed the stability of MoS_2_ nanosheets dispersion in aqueous solutions and revealed that the MoS_2_ was a two-dimensional thin nanosheet (Figure S1A). Figure S1B demonstrated that the MoS_2_ nanosheets possessed their two typical absorption peaks at around 607 and 665 nm by UV-visible absorption spectrum, which is consistent with the reported results [25]. The AFM equipment not mentioned in 2.2 image was also recorded to characterize the size of MoS_2_. As shown in Figure S1C, the AFM image displayed that the height of the MoS_2_ sheet was about 2.7 nm thick (inset in Figure S1C), indicating that the MoS_2_ is a few-layer nanosheet. The characterization of the MoS_2_ nanosheets was further confirmed by dynamic light scattering (DLS). The result indicated that the lateral dimensions of most of MoS_2_ nanosheets were about 100 to 400 nm, suggesting that the nanosheets had a high polydispersity [27,28].

### 3.2. Feasilbility of the Assay 

**Figure 1:**
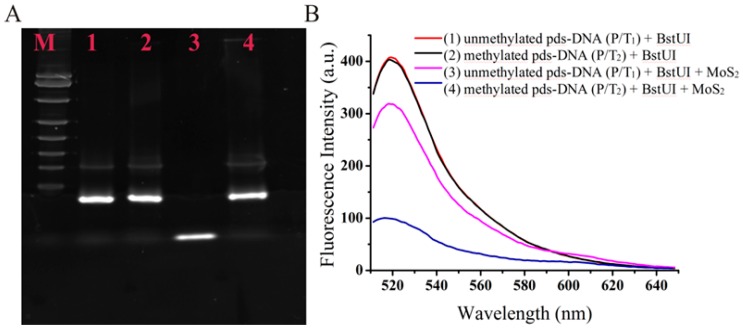
(**A**) Gel image about the methylation-sensitive cleaved process of BstUI in non-denaturing polyacrylamide gel electrophoresis. Lane M: DNA marker; Lane 1: unmethylated pds-DNA (P/T_1_); Lane 2: methylated pds-DNA (P/T_2_); Lane 3: unmethylated pds-DNA (P/T_1_) + BstUI; Lane 4: methylated pds-DNA (P/T_2_) + BstUI. (**B**) Fluorescence emission spectra of the DNA detection and methylation analysis strategy under different conditions: (1) unmethylated pds-DNA (P/T_1_); (2) methylated pds-DNA (P/T_2_); (3) unmethylated pds-DNA (P/T_1_) + BstUI + MoS_2_; (4) methylated pds-DNA (P/T_2_) + BstUI + MoS_2_.

The principle of the method was outlined in Scheme 1. In this system, BstUI specifically cleaves the residue of unmethylated pds-DNA (P/T_1_) containing the human methylation specific site 5’-CGCG-3’, and a FAM-labeled complementary DNA is released, while the methylated pds-DNA (P/T_2_) could not be digested by BstUI [29,30]. To validate the methylation-sensitive cleavage process of BstUI, the PAGE test was performed. As shown in Figure 1A, in the absence of BstUI, both the bands of unmethylated pds-DNA (P/T_1_, line 1) and methylated pds-DNA (P/T_2_, line 2) were identical. When the BstUI was added into pds-DNAs solutions, the band situation of unmethylated pds-DNA (P/T_1_, line 3) was lower than those of the above-mentioned cases, showing that the unmethylated pds-DNA was cleaved into shorter chains by BstUI endonuclease.

Nevertheless, there was no change in the band of methylated pds-DNA (P/T_2_, line 4), suggesting that the cleavage of BstUI endonuclease was blocked by methylation. At the same time, little difference of fluorescence intensities between unmethylated pds-DNA (P/T_1_, Figure 1B curve 1) and methylated pds-DNA (P/T_2_, Figure 1B curve 2) was observed. Upon addition of MoS_2_ nanosheets, the methylated pds-DNA (P/T_2_) containing the single-stranded was tightly adsorbed by MoS_2_ nanosheets and the fluorescence intensity of P/T_2_ was sharply quenched (Figure 1B curve 4). But unmethylated pds-DNA (P/T_1_) cleaved by BstUI just slightly interacted with MoS_2_ nanosheet in the form of totally complementary DNA, and exhibited a little lower fluorescence signal than that in the absence of MoS_2_ (Figure 1B curve 3). The accordance of fluorescence spectroscopy experiment with the aforementioned gel analysis successfully demonstrates that the designed sensing system is feasible.

### 3.3. Optimization of Assay Conditions 

To obtain the best sensing performance, the optimal conditions of experimental parameters, including MoS_2_ concentration, concentration of BstUI endonuclease and cleavage time, were evaluated by comparing the relative fluorescence change. The relative fluorescence change was expressed as the signal difference value ΔF.

ΔF = F_1_ − F_2_, where F_1_ and F_2_ are fluorescence intensities of the system where the primer FAM-probe are hybridized with its complementary unmethylated (T_1_) and methylated (T_2_) target DNA, respectively.

**Figure 2:**
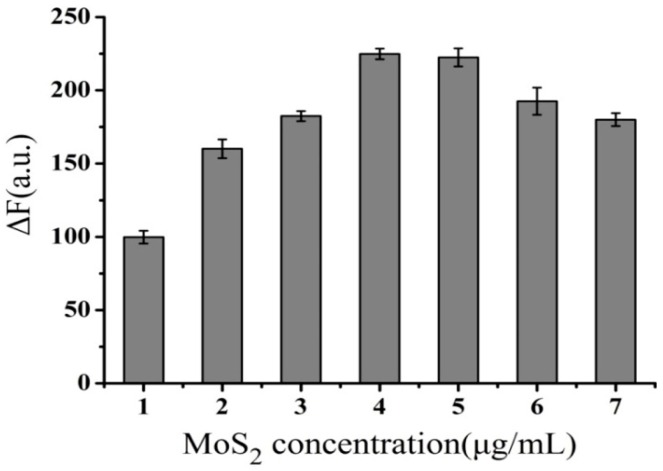
Dependence of the relative fluorescence change (∆F = F_1_ − F_2_) on different concentrations of MoS_2_ nanosheets. Error bars represent the standard deviation of three experiments.

The MoS_2_ concentration played a decisive role in distinguishing between the partial duplex DNA (pds-DNA) and the double-stranded DNA (ds-DNA). As shown in Figure 2, in the concentration range of MoS_2_ at 1.0–4.0 μg/mL, the ΔF increased significantly as the concentration of MoS_2_ increased. However, when the concentration of MoS_2_ exceeded 4.0 μg/mL or higher, the ΔF decreased, indicating that the MoS_2_ of high concentration would cause an excessive quenching effect on the cleavage-produced FAM-labeled double-stranded DNA. According to the above results, a concentration of MoS_2_ at 4.0 μg/mL was selected for the following analysis experiments.

In addition, the concentration of BstUI endonuclease and cleavage time also affect the sensing system. As can be seen in Figure S2A, as the concentration of BstUI increased, the ΔF was enhanced and reached a maximum at 20 U/mL. Similarly, as depicted in Figure S2B, the ΔF increased and reached a plateau phase when the cleavage time was 2 h. As a result, 20 U/mL BstUI endonuclease and 2 h cleavage time were selected as the optimum conditions of BstUI for the following analysis experiments.

### 3.4. Kinetic Behavior 

**Figure 3:**
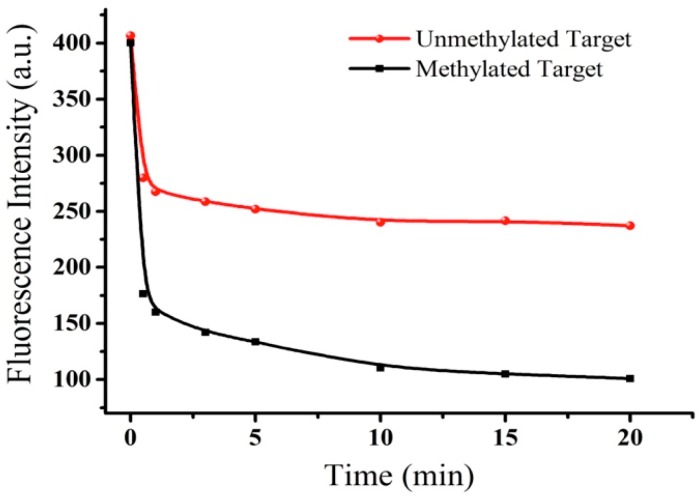
The kinetic behavior of FAM-probe fluorescence quenching in the presence of MoS_2_ with the unmethylated target (red) and methylated target (blank), respectively.

The kinetic behavior of MoS_2_ fluorescence quenching was investigated as well by monitoring the fluorescence intensity as a function of time. Figure 3 shows the fluorescence quenching of P/T_1_ (curve red) and P/T_2_ (curve blank) after digested in the presence of MoS_2_ as a function of incubation time. Upon addition of MoS_2_ (4 μg/mL) into the solutions, the quenching was found to be very fast. After that, the fluorescence intensity of P/T_1_ decreased slightly and reached a stable signal for 10 min. Similarly, the fluorescence intensity of P/T_2_ decreased sharply in the first 30 s and almost reached the equilibrium within 10 min. In other words, the relative fluorescence change of both P/T_1_ and P/T_2_ achieved maximum when the quenching time was at 10 min.

### 3.5. Detection of Target DNA 

**Figure 4:**
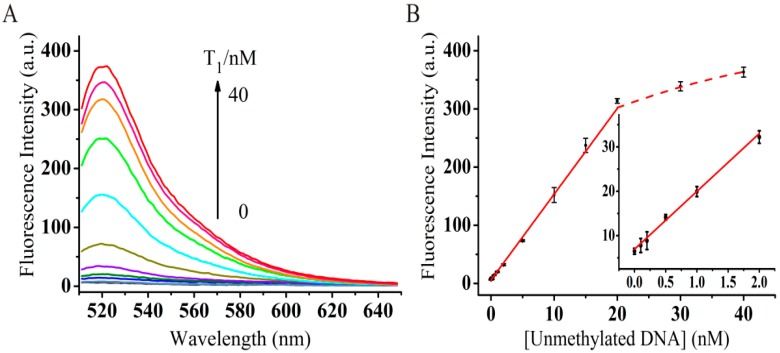
(**A**) Fluorescence spectra of the hybridization of 20 nM probe DNA with various concentrations of T_1_ (0, 0.1, 0.2, 0.5, 1, 2, 5, 10, 15, 20, 30 and 40 nM); (**B**) The linear relationship of F (Y) with various DNA concentrations (X) ranging from 0 to 40 nM. The inset shows linear responses to low concentrations of T_1_ ranging from 0 to 2 nM. Error bars represent the standard deviation of three experiments.

Firstly, based on the high fluorescence quenching property and discrimination ability between ss-DNA and ds-DNA of MoS_2_ [19], the unmethylated target DNA (T_1_) (a fragment of P16 promoter) could be detected in this homogeneous sensing system. Figure 4A exhibited the fluorescence spectra of 20 nM FAM-probe DNA (P) in the presence of different concentrations of unmethylated target DNA (T_1_) from 0 to 40 nM under the optimized conditions. It is noted that fluorescence could still increase as the concentration of T_1_ exceeded that of FAM-probe DNA (P). That’s because that the redundant T_1_ has a stronger interaction with MoS_2_ compared to the cleaved double-stranded DNA of P/T_1_, and replaces the few adsorbed cleaved ds-DNA on MoS_2_ [19].

According to the relative calibration curve (Figure 4B), the MoS_2_-based DNA sensor revealed a linear response in the range from 0 to 20 nM with the calibration equation is Y = 5.81X + 14.75 (Y represents the fluorescence intensity and X represents T_1_ concentrations, R^2^ = 0.9946). The detection limit is 140 pM obtained in terms of 3 times deviation of blank sample, which is at the same magnitude with the previously reported nanomaterials-based fluorescent assay [20,31].

In order to investigate the specificity of detection of target DNA, one base mismatched DNA (M), non-complementary DNA (N), and unmethylated target DNA (T_1_) were employed, respectively. It was seen that the unmethylated DNA (T_1_, a) possessed the highest fluorescence response (Figure S3). In contrast, the fluorescence intensity of M (b) and N (c) decreased greatly, only a slightly higher than that of blank control (d). The results indicate that the MoS_2_ nanosheet-based fluorescence biosensor has a satisfactory specificity.

### 3.6. Quantitative Analysis of DNA Methylation 

Promoter region of p16 is known to be differentially methylated in a variety of physiological states. Therefore, we use it as an example to check the efficacy of our sensor in quantitiative analysis of DNA methylation. We prepared 20 nM probe DNA (P) to hybridize with the artificial mixtures consisting of 20 nM methylated and unmethylated target DNA at different ratios, 0, 5%, 10%, 20%, 30%, 40%, 60%, 80%, and 100% methylated target DNA. The methylation level of target DNA at 5’-CGCG-3’ could be evaluated from the formula:

Methylation levels % = M / (U + M) × 100 %
(1)

**Figure 5:**
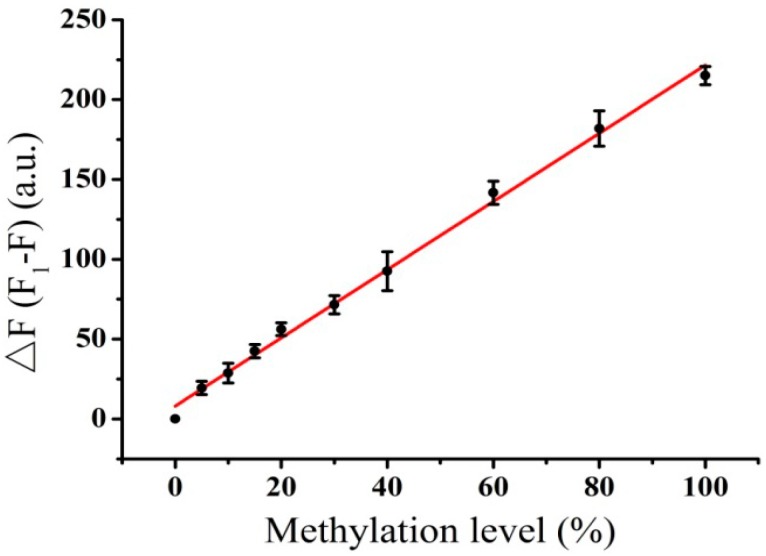
The linear relationship between ∆F (Y) and methylation level (X) ranging from 0% to 100% (5%, 10%, 15%, 20%, 30%, 40%, 60%, 80%, and 100%). Error bars represent the standard deviation of three experiments.

Under the optimized conditions, experiments were carried out by adding the mixtures with increasing methylation levels into the proposed MoS_2_-based sensing system to examine whether the relative fluorescence change (ΔF, ΔF = F_1_ – F, where F_1_ and F are fluorescence intensities of the system where the primer FAM-probe is hybridized with the unmethylated DNA (T_1_) and mixtures of different methylation proportions target DNA (T), respectively) could be used for methylation level analysis. As presented in Figure 5, the relative fluorescence change (ΔF) increased with the increasing DNA methylation level, indicating a linear correlation in the DNA methylation level range from 0 to 100%. The calibration equation is Y = 2.14X + 8.09 (Y represents ΔF and X represents methylation level, R^2^ = 0.9956). The detection limit was calculated to be 1.0% based on LOD = 3 σ/S (σ is the standard deviation of the intercept and S is the slope of the calibration graph, n = 3). The result was close to that of the previously reported methylation level analysis [5,14].

The reproducibility of the proposed biosensing system is essential for an assay’s protential in practical application. The reproducibility was assessed by calculating the relative standard deviations (RSD) and measured in different days at the identical experimental conditions. The RSD (n = 3) with the DNA detection were 4.4%, 2.0%, 1.3% at 0.5 nM, 5 nM and 20 nM Unmethylated DNA (T_1_), respectively. And the RSD (n = 3) with the methylation analysis were 7.2%, 5.1%, 2.7% at the DNA methylation level of 20%, 60% and 100%, respectively. These results demonstrate that the proposed biosensing system possesses optimistic reproducibility.

## 4. Conclusions 

In summary, we developed a simple nanobiosensing platform for DNA-methylation analysis with MoS_2_ nanosheets and demonstrated its efficacy on p16 promoter in homogenous solution. Unlike conventional DNA-methylation detection technologies, our method does not rely on bisulfite and PCR, providing a straightforward fluorescence readout of methylation level within 3 h. Without any amplification strategy, the assay could distinguish as low as 1% methylation level in the mixtures with excellent reproducibility. The biosensing platform could also be used to detect DNA with a detection limit of 140 pM with high sensitivity. Thus, a homogeneous quantitative analysis of DNA methylation was provided with short-time, easy operation, as well as relative good sensitivity. The results indicate that such nanobiosensor is promising in nucleic acid detection, particularly quantitative analysis of DNA methylation at the point of care.

## Figures and Tables

**Table 1:** Used DNA sequences.

Oligonucleotides	Gene Sequences (5′–3′)
FAM-Probe DNA (P)	FAM-GAC CCC GGG CCG CGG CCG TGG
Unmethylated target DNA (T_1_)	AGC AGC ATG GAG CCT TCG GCT GAC TGG CTG GCC ACG GCC GCG GCC CGG GGT C
Methylated target DNA (T_2_)	AGC AGC ATG GAG CCT TCG GCT GAC TGG CTG GCC ACG GCmC GmCG GCC CGG GGT C
One base mismatched DNA (M)	AGC AGC ATG GAG CCT TCG GCT GAC TGG CTG GCC ACG GCC TCG GCC CGG GGT C
Non-complementary DNA (N)	CTT GAT ATT CGG CAC ATA GTC CTG GGA GAG ACC GGC GCA CAG AGG AAG AGA A

## Supplementary Materials

The following are available online at http://www.mdpi.com/1424-8220/16/10/1561/s1, **Figure S1****:** Characterization of the MoS_2_ nanosheets. (**A**) TEM image of MoS_2_; (**B**) UV-visible absorption spectrum of MoS_2_; (**C**) AFM image and height profile (inset) of MoS_2_; (**D**) The lateral dimension distribution of MoS_2_. **Figure S2**: Effect of different BstUI endonuclease concentrations (**A**) and cleavage reaction time (**B**) on ΔF. Error bars show the standard deviation of three experiments. **Figure S3**: The specificity of detection of target DNA. Fluorescence emission spectra in the presence of (**a**) complementary unmethylated target DNA (T_1_), (**b**) one-base mismatched DNA (M), (**c**) noncomplementary DNA (N) and (**d**) blank. The inset shows the histogram corresponds to the fluorescence spectra in fluorescence emission spectra. Error bars represent the standard deviation of three experiments.

## Abbreviations

MoS_2_	molybdenum disulfide
pds-DNA	partial duplex-deoxyribonucleic acid
ss-DNA	single stranded-deoxyribonucleic acid
ds-DNA	double stranded-deoxyribonucleic acid
Tris	Tris(hydroxylmethyl)aminom-ethan
NaCl	sodium chloride
MgCl_2_	magnesium chloride
TEM	transmission electron microscopy
AFM	atomic force microscopy
DLS	dynamic light scattering
UV	ultraviolet
PAGE	polyacrylamide gel electrophoresis
