# Supplementary material for: A MoS2 Nanosheet-Based Fluorescence Biosensor for Simple and Quantitative Analysis of DNA Methylation
Source: Sensors (Basel). 2016 Sep 22;16(10):1561. doi: 10.3390/s16101561 (PMC5087350; doi:10.3390/s16101561)
Supplement: Supplementary file 1 [file sensors-16-01561-s001.pdf]

Supplementary Materials: A MoS_2 Nanosheet-Based Fluorescence Biosensor for Simple and Quantitative Analysis of DNA Methylation

Le Xiao, Li Xu, Chuan Gao, Yulin Zhang, Qunfeng Yao and Guo-Jun Zhang

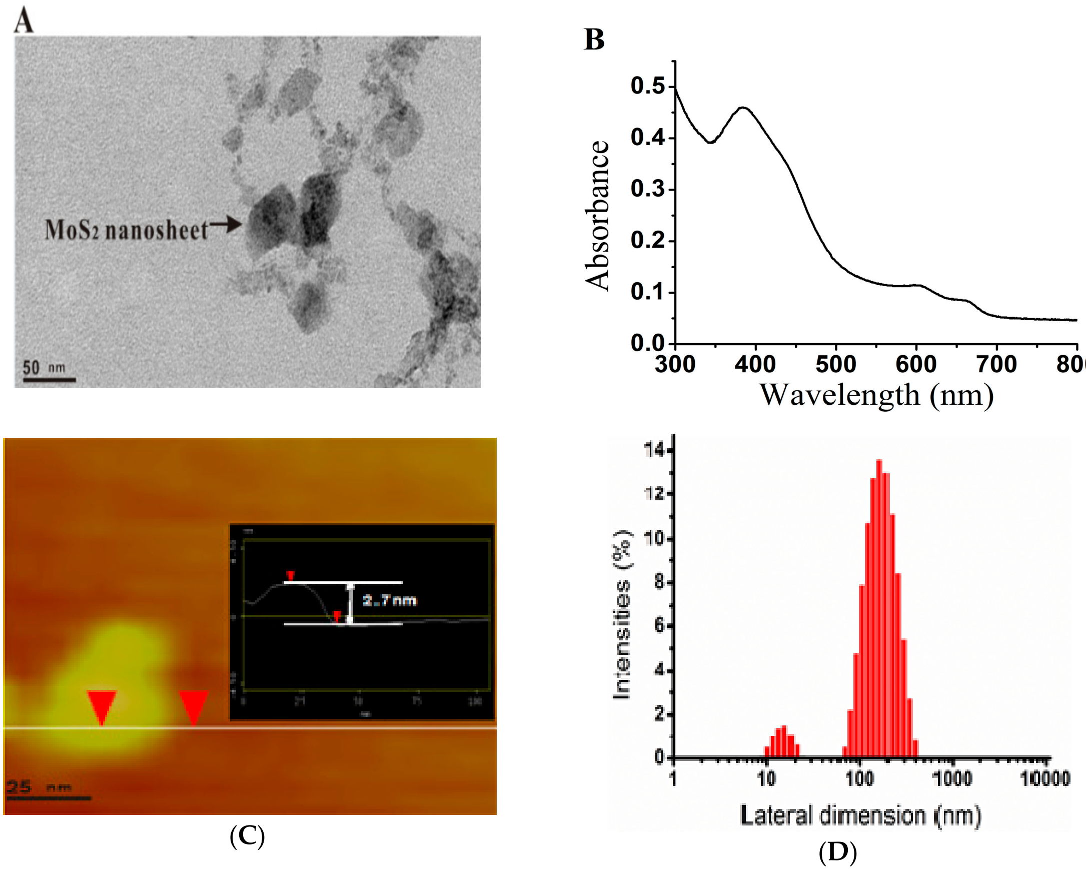

Figure. S1. Characterization of the MoS_2 nanosheets. (**A**) TEM image of MoS_2; (**B**) UV-visible absorption spectrum of MoS_2; (**C**) AFM image and height profile (inset) of MoS_2; (**D**) The lateral dimension distribution of MoS_2.

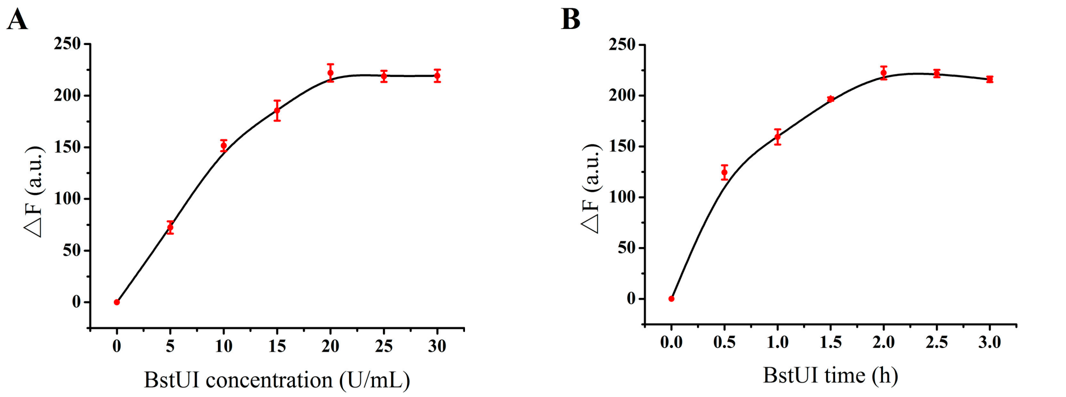

Figure. S2. Effect of different BstUI endonuclease concentrations (**A**) and cleavage reaction time (**B**) on ΔF. Error bars show the standard deviation of three experiments.

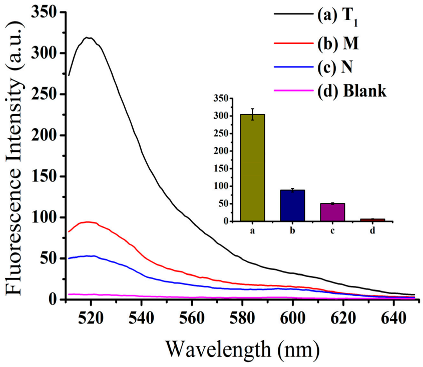

Figure. S3. The specificity of detection of target DNA. Fluorescence emission spectra in the presence of (**a**) complementary unmethylated target DNA (T_1); (**b**) one-base mismatched DNA (M); (**c**) noncomplementary DNA (N) and (**d**) blank. The inset shows the histogram corresponds to the fluorescence spectra in fluorescence emission spectra. Error bars represent the standard deviation of three experiments.
